# Characteristics of glucose metabolism indexes and continuous glucose monitoring system (CGMS) in patients with insulinoma

**DOI:** 10.1186/s13098-017-0215-3

**Published:** 2017-03-14

**Authors:** Weijun Gu, Yixin Liu, Hongyan Liu, Guoqing Yang, Qinghua Guo, Jin Du, Nan Jin, Li Zang, Zhaohui Lv, Jianming Ba, Yiming Mu, Jingtao Dou

**Affiliations:** 1 Department of Endocrinology, Chinese PLA General Hospital, No. 28 Fuxing Road, Haidian District, Beijing, 100853 People’s Republic of China; 2 Department of Internal Medicine, The Affiliated Hospital of Institute of Aviation Medicine, Beijing, 100089 People’s Republic of China

**Keywords:** Insulinoma, Continuous glucose monitoring, Hypoglycemia, Glucose variability

## Abstract

**Aims:**

Analyze the clinical applicability of glucose metabolism indexes and continuous glucose monitoring data on the qualitative diagnosis of insulinoma.

**Methods:**

Involve 22 patients with insulinoma (insulinoma group), 11 patients with hypoglycemia (hypoglycemia group) and 31 people with normal glucose tolerance (control group). HbA1c, fasting blood glucose (FBG), insulin (FINS) and C-peptide (FCP) was tested. Using CGMS to monitor the blood glucose for three consecutive days and selecting the monitoring data of 24 h thereof, figuring out, with the aid of EasyGV Version 9.0, the mean glucose (MG), the standard deviation (SD) of blood glucose, CONGA (continuous overall net glycemic action), J-Index, LI (Lability Index), LBGI (Low Blood Glucose Index), HBGI (High Blood Glucose Index), GRADE (glycaemic risk assessment diabetes equation), MAGE (mean aplitude of glycaemic excursions), M value, MAG (mean absolute glucose).

**Results:**

(1) FBG and LBG of insulinoma group are lower than those of control group and those of hypoglycemia group while FINS and FCP of insulinoma group are markedly higher than those of the other two groups; (2) the MG and CONGA of insulinoma group are lower than those of control group and its indexes like ST, LI, LBGI, GRADE, MAGE, M value and MAG are higher than those of control group; there are differences between the indexes of insulinoma group and those of hypoglycemia group in CONGA (lower than that of hypoglycemia group), LBGI (higher than that of hypoglycemia group), and M value (higher than that of hypoglycemia group). By drawing the ROC curve and calculating Youden index, the cut-off values of LBGI, M value, CONGA are respectively as 4.06, 7.79, 4.38, and the best index of differential diagnosis is LBGI.

**Conclusion:**

Continuous glucose monitoring data can be used to diagnose insulinoma and blood glucose fluctuation indicators such as LBGI, M value, CONGA might be useful to identify insulinoma.

## Background

Insulinoma is the most common functional islet cell tumor of which the most prominent characteristic is the severe status of hypoglycemia caused by improper insulin secretion. It is a common cause of organic hypoglycemia of which the symptoms are diverse and there is a lack of uniqueness [1]. If the nervous system gradually gets accustomed to the chronical hypoglycemia, there will appear asymptomatic hypoglycemia [2], which shall bring about high rates of missed diagnosis and misdiagnosis. More than 50% of the patients are diagnosed to have suffered from it for over five years [3].

The continuous glucose monitoring system is capable of providing the information about blood glucose in all day consecutively and has unique advantages in assessing the fluctuation of blood glucose and in discovering hypoglycemia. Combining with other clinical glucose metabolism indexes, it can be used to discuss how useful it can be to the qualitative diagnosis of insulinoma.

## Subjects and methods

### Data and methods

#### Subjects

The study has selected 22 patients who, hospitalized in the endocrinology wards in 301 hospital from May 2011 to April 2014, were diagnosed with insulinoma and examined by the continuous glucose monitoring system (the insulinoma group), 8 of the patients are men and 14 women with their age ranging from 22 to 71. All of them underwent surgery and pathologically confirmed insulinoma. 11 patients with non-islet cell tumor who were hospitalized because of reactive hypoglycemia during the same period (the hypoglycemia group), there are six men and five women ranging from 39 to 73 years old. Patients with diabetes, any other organic pathology or drug-induced hypoglycemia were excluded; reactive hypoglycemia is defined when recurrent episodes of symptomatic hypoglycemia occur 2–4 h after a high carbohydrate meal (or oral glucose load),and the result of 72-h fasting test is negative; 31 people with normal glucose metabolism (the control group) on the basis of the diagnosis standards of diabetes under WHO and for the purpose of the glucose tolerance experiment, among whom there 15 men and 16 women ranging from 20 to 70 years old. All subjects are free of any severe hepatorenal damage or other serious stress state such as cardiac insufficiency, trauma, pregnancy, tumor, and infectious diseases. The study was conducted in accordance with the principles of the Declaration of Helsinki and approved by the Ethics Committee of the Chinese PLA General Hospital. Written informed consent was obtained from each patient before enrollment.

#### Methods

##### Collection of clinical data

Take a detailed record of all subjects’ age, sex, BMI, course of disease, as well as the biochemical indicators including HbA1c, fasting blood glucose, uric acid, and blood lipid.

##### Continuous glucose monitoring system and collection of data

All monitoring work is developed with the CGMS from Medtronic Inc. Implant the probe of the CGMS into the abdominal subcutaneous tissue and monitor the glucose concentration of the interstitial fluid of subcutaneous tissues in 72 h. Take down a MG every 5 min and 288 blood glucose values within 24 h. In the meanwhile, use the Bai'anyi blood glucometer from Bayer company to determine the blood glucose of the fingertip capillaries for at least 4 times a day and enter the results into CGMS for correcting. Strenuous exercise or shower is not allowed during the period of glucose monitoring.

##### Assessment of glucose level and the fluctuation parameters of it

The CGMS software can offer the amplitude of glucose fluctuation (the highest and lowest numbers of blood glucose), the mean blood glucose (MBG), the standard deviation of blood glucose (SD) and the bigger the SD is, the bigger the difference there will be between the average and mean blood glucose, and the bigger the amplitude of glucose fluctuation will be; by setting the capping and floor of the blood glucose (here we assume the capping is 10.0 mmol/L and the floor 2.8 mmol/L), the proportion of time distribution beyond the capping/floor as well as the residence time can be obtained; the times of blood glucose fluctuation in all day and the times of hyperglycemia/hypoglycemia fluctuation. The software counts the blood glucose fluctuating for more half an hour as one time. In addition, calculating the mean glucose (MG), the standard deviation of glucose (SD), CONGA (continuous overall net glycemia action), J-Index, LI (Liability Index), LBGI (Low Blood Glucose Index), HBGI (High Blood Glucose Index), GRADE (glycaemic risk assessment diabetes equation), MAGE (mean aplitude of glycaemic excursions), M value, MAG (mean absolute glucose).

#### Statistical methods

The normal distributions of the measurement data are represented by x ± s, the inter-group comparison is analyzed by *t* test or variance, the comparison of non-normal distribution data adopts the non-parametric test. ROC curves are used to compare the diagnostic performance of some indexes. Statistical treatment of the data adopts SPSS19.O with P < 0.05 as the statistical deviation.

## Results

### Comparison of the general data of each group

The general data of the insulinoma group, the hypoglycemia group and the control group (see Table 1). The inter-group differences of the course of diseases and the BMI are of statistical significance, and the differences of other indexes (age, blood pressure, blood lipid, uric acid) have no such significance. Patients in the insulinoma group have longer course of disease than patients in the hypoglycemia group do, with the longest course reaching 20 years, and their BMI are higher than patients in the hypoglycemia group and people in the control group with the average value of 28.44 ± 4.49 kg/m^2^.

**Table 1:** Comparison of general data of patients in three groups

Variables	Insulinoma group (44)	Hypoglycemia group (26)	Control group (31)	P value
Sex (M/F)	16/28	14/12	15/16	
Age	50.59 ± 13.2	55.64 ± 12.6	53.23 ± 11.9	0.302
Course	6.43 ± 6.94*	1.62 ± 2.24	–	0.006
BMI	28.44 ± 4.49*	24.54 ± 3.75	22.7 ± 2.6	0.019
SBP	129.32 ± 14.35	138.0 ± 14.21	133.0 ± 14.18	0.11
DBP	80.64 ± 10.45	77.91 ± 8.32	78.0 ± 7.45	0.458
UA	303.02 ± 63.77	273.87 ± 82.9	282.32 ± 9.52	0.30
TG	1.41 ± 1.05	1.20 ± 0.50	1.15 ± 0.87	0.55
CHO	4.16 ± 0.92	4.16 ± 0.80	4.46 ± 0.8	0.99
HDL	1.16 ± 0.41	1.30 ± 0.55	1.41 ± 0.61	0.46
LDL	2.46 ± 0.8	2.4 ± 0.68	2.43 ± 0.78	0.85

*BMI* Body Mass Index, *SBP* systolic blood pressure, *DBP* diastolic blood pressure, *UA* uric acid, *TG* triglycerides, *CHO* total cholesterol, *HDL* high-density lipoprotein cholesterol, *LDL* low-density lipoprotein cholesterol

*P < 0.05

### Comparison of glucose metabolism indexes of three groups

Compare the glucose metabolism indexes of three groups and find out that there are marked differences in the fasting blood glucose, the lowest blood glucose in all day, the fasting insulin and the fasting C-peptide. Make pairwise comparison of those indexes and discover that the fasting blood glucose and the lowest blood glucose in all day of the insulinoma group are lower than those of the control group and the hypoglycemia group, while the fasting insulin and the fasting C-peptide are higher than those of the other two groups. All the differences are of statistical significance (see Table 2).

**Table 2:** Comparison of glucose metabolism indexes of three groups

Variables	Insulinoma group (n = 44)	Hypoglycemia group (n = 26)	Control group (n = 31)	F value	P value
HBG (mmol/L)	9.00 ± 3.10	9.65 ± 4.47	8.27 ± 1.51	0.95	0.628
LBG (mmol/L)*	2.76 ± 0.92	3.93 ± 0.75	4.67 ± 0.63	9.74	0.001
MBG (mmol/L)	5.10 ± 1.20	5.99 ± 1.63	5.83 ± 0.66	2.86	0.085
SD	1.46 ± 0.79	1.25 ± 1.15	0.92 ± 0.28	1.03	0.526
HbA1c (%)	4.97 ± 0.50	5.27 ± 0.39	5.52 ± 0.21	2.14	0.101
FBG (mmol/L)*	3.37 ± 1.29	4.83 ± 0.40	4.74 ± 0.37	14.72	0.000
FINS (mU/L)*	22.63 ± 15.09	8.04 ± 6.19	8.60 ± 3.60	10.72	0.000
FCP (ng/mL)*	3.17 ± 1.18	2.17 ± 1.04	2.23 ± 0.69	5.07	0.011
IRI*	0.45 ± 0.36	0.09 ± 0.07	0.10 ± 0.05	12.80	0.000

*HBG* high blood glucose, *LBG* low blood glucose, *MBG* mean blood glucose, *SD* standard deviation, *HbA1c* glalycosylated hemoglobin A1c, *FBG* fasting blood glucose, *FINS* fasting insulin, *FCP* fasting C-peptide, *IRI* Insulin Release Index

*P < 0.05

### Comparison of occurrence of hypoglycemia of patients in the insulinoma group and patients in the hypoglycemia group

Comparing with patients in the hypoglycemia group, the patients with insulinoma tend to suffer more hypoglycemia, with longer course and more severe consequences: the insulinoma group has more hypoglycemia fluctuations, lower number of the lowest blood glucose, more percentage beyond the floor set for glucose and longer course. Within 24 h, there 11 patients (50.0%) in the insulinoma group suffer hypoglycemia, averagely every 110 min the blood glucose of one person is less than 2.8 mmol/L, accounting for 7.67%; whereas in the hypoglycemia group there are only two people (15.4%) suffering hypoglycemia, one person every 20 min (see Table 3).

**Table 3:** Comparison of occurrence of hypoglycemia of patients in the insulinoma group and patients in the hypoglycemia group

Variables	Insulinoma group (n = 44)	Hypoglycemia group (n = 26)	P value
Times of glucose fluctuation*	2.68 ± 2.51	0.45 ± 0.93	0.001
Times of hypoglycemia fluctuation*	2.23 ± 2.18	0 ± 0.00	0.000
Lowest glucose in all day*	2.76 ± 0.92	3.93 ± 0.75	0.001
Lowest glucose at night*	3.17 ± 1.17	4.6 ± 0.94	0.001
Proportion beyond the capping	2.23 ± 5.54	3.55 ± 8.43	0.593
Proportion beyond the floor*	7.56 ± 10.37	0.01 ± 0.03	0.003
Time beyond the capping	32.05 ± 79	45.45 ± 104.37	0.683
Time beyond the floor*	108.86 ± 149.7	0 ± 0.00	0.003

*P < 0.05

### Comparison of post-CGMS glucose variation indexes of patients in three groups

The comparison of inter-group skewed distribution data adopts the rank-sum test and there are remarkable differences among the groups of other indexes with the exception of J-Index and HBGI (see Table 4). Then make pairwise comparison of indexes with inter-group differences (see Tables 5, 6). Comparing with the control group in the rank means of each index, the insulinoma group has lower MG and GONGA, higher SD, LI, LBGI, GRADE, MAGE, M value, and MAG (P < 0.0167); comparing with the hypoglycemia group, the MG is similar, but the insulinoma group has lower CONGA and higher LBGI and MAG (P < 0.0167).

**Table 4:** Comparison of post-CGMS glucose variation indexes of patients in three groups

Variables	Insulinoma group (n = 44)	Hypoglycemia group (n = 26)	Control group (n = 31)	Chi square	P value
MBG*	23.59	34.62	40.06	9.53	0.009
SD*	42.23	34.31	26.97	8.16	0.017
CONGA*	20.00	37.62	41.35	16.67	0.000
LI*	45.32	31.46	25.97	13.26	0.001
J-Index	29.59	36.38	35.06	1.41	0.494
LBGI*	49.68	31.38	22.90	25.24	0.000
HBGI	34.25	37.92	31.11	1.20	0.548
GRADE*	46.61	30.77	25.34	16.15	0.000
MAGE*	41.32	33.17	27.03	7.35	0.025
M value*	49.09	33.92	22.26	25.15	0.000
MAG*	49.36	34.85	21.68	26.85	0.000

*MBG* mean blood glucose, *SD* standard deviation, *CONGA* continuous overall net glycemic action, *LI* lability index, *LBGI* Low Blood Glucose Index, *HBGI* High Blood Glucose Index, *GRADE* glycaemic risk assessment diabetes equation, *MAGE* mean aplitude of glycaemic excursions, *MAG* mean absolute glucose

*P < 0.05

**Table 5:** Pairwise comparison of CGMS indexes

Variables	Insulinoma group	Control group	Z value	P value
MBG*	19.27	32.48	−3.069	0.002
SD*	34.41	21.74	−2.942	0.003
CONGA*	16.95	34.13	−3.989	0.000
LI*	36.27	20.42	−3.682	0.000
LBGI*	39.23	18.32	−4.856	0.000
GRADE*	37.27	19.71	−4.084	0.000
MAGE*	34.00	22.03	−2.780	0.005
M value*	39.23	18.32	−4.856	0.000
MAG*	40.00	17.77	−5.163	0.000

*MBG* mean blood glucose, *SD* standard deviation, CONGA continuous overall net glycemic action, *LI* Lability Index, *LBGI* Low Blood Glucose Index, *GRADE* glycaemic risk assessment diabetes equation, *MAGE* mean aplitude of glycaemic excursions, *MAG* mean absolute glucose

*P < 0.05

**Table 6:** Pairwise comparison of CGMS indexes

Variables	Insulinoma group	Hypoglycemia group	Z value	P value
MBG	15.82	21.69	−1.639	0.101
SD	19.32	15.77	−0.990	0.322
CONGA*	14.55	23.85	−2.595	0.009
LI	20.55	13.69	−1.912	0.056
LBGI*	21.95	11.31	−2.970	0.003
GRADE	20.84	13.19	−2.136	0.033
MAGE	18.82	15.08	−1.045	0.296
M value*	21.36	12.31	−2.526	0.012
MAG	20.86	13.15	−2.151	0.031

*MBG* mean blood glucose, *SD* standard deviation, CONGA continuous overall net glycemic action, *LI* Lability Index, *LBGI* Low Blood Glucose Index, *GRADE* glycaemic risk assessment diabetes equation, *MAGE* mean aplitude of glycaemic excursions, *MAG* mean absolute glucose

*P < 0.05

### Diagnostic indexes and the cut-off point for insulinoma

**Fig. 1:**
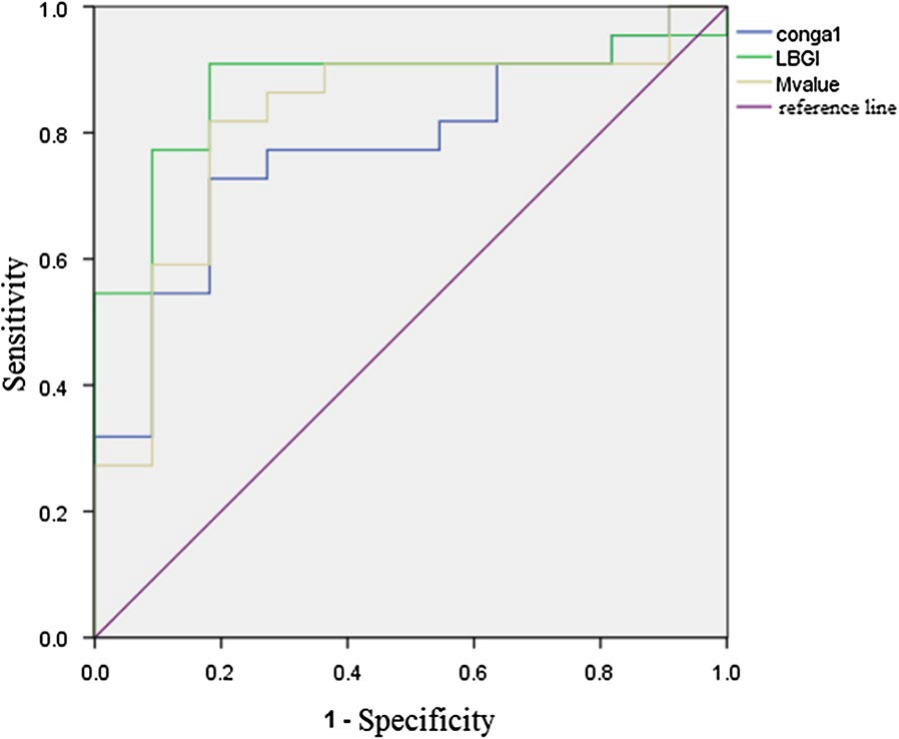
Roc curve of LBGI, M value, CONGA for insulinoma diagnosis. *MBG* mean blood glucose, *CONGA* continuous overall net glycemic action, *LBGI* Low Blood Glucose Index

There is significant difference in the glucose variation indexes, CONGA, LBGI, M value between the two groups, so these three indexes are considered as examined variable. Area under the ROC curve of LBGI, M, CONGA is 87.2, 81.8 and 77.3%, respectively. The LBGI cut-off point is 4.06, the corresponding sensitivity (with 95% CI) is 90.9%, specificity (with 95% CI) 81.8%, with the Youden 73%; the M value cut-off point is 7.79, the corresponding sensitivity (with 95% CI) and specificity (with 95% CI) are both 81.8%, with the Youden 55%; the CONGA cut-off point is 4.38, the corresponding sensitivity (with 95% CI) is 72.7%, specificity (with 95% CI) 81.8%, with the Youden 55%. Based on the Youden and area under the ROC curve value, LBGI seems to be of the highest accuracy (see Fig. 1).

## Discussion

Insulinoma, first described by Graham in 1927, originates from islet β cells, also known as islet β cell tumors, and has an occurrence rate of around 4/1,000,000. Unlike other pancreatic neuroendocrine tumors, most insulinomas are benign, the malignant ones only account for 5–10% [4], and are commonly seen in single adenomas. Insulinoma often occurs to people from 40 to 50 years old with similar occurrences between men and women.

The diagnosis of insulinoma consists of the qualitative diagnosis and the topographic diagnosis. At present, the qualitative diagnosis, of which the main means is laboratory examination, prevails in confirming the diagnosis. The laboratory examination, including determining the blood glucose of hypoglycemia, determining simultaneously the serum insulin and the C-peptide, and carrying out starvation test, aims at confirming the existence of hypoglycemia and that it is caused by the improper secretion of endogenous insulin, that is, when the plasma glucose concentration ≤2.80 mmol/L, the insulin concentration >25 mIU/L, the Insulin Release Index (IRI) >0.3. The topographic diagnosis is dependent on the imaging examination and due to the small size of the tumor and the difficulty in diagnosis, it is mainly used to instruct the operations.

Comparing the general information of all subjects, it can be found out that for patients in the insulinoma group, the time from the onset of the disease to the confirmation of diagnosis is significantly longer than that for patients in the hypoglycemia group (P < 0.01). In order to prevent the incidence of hypoglycemia during the long course, the patients with insulinoma frequently increase their diets and thus cause the rise of the BMI. Hence is the statistical significance in the differences against the patients in the hypoglycemia and control groups (P < 0.01).

The study demonstrates that the fasting blood glucose of patients with insulinoma is lower than that of patients with hypoglycemia, and the fasting insulin, the fasting C-peptide, and the fasting IRI of patients with insulinoma are higher than those of patients with hypoglycemia. The differences are of statistical significance. Similar to the findings of the study, Han et al. [5], in comparing the glucose metabolism indexes of patients with hypoglycemia caused by various reasons, also discover that in the OGTT (oral glucose tolerance test), the differences of the fasting blood glucose, the fasting IRI and C-peptide indexes among the insulinoma group, the functional hypoglycemia group, the impaired glucose tolerance or diabetes group are of statistical significance. The crucial step in the current qualitative diagnosis of insulinoma is to monitor the hypoglycemia and simultaneously determine the IRI >0.3 when the plasma glucose <2.8 mmol/L. However, there are studies indicating that the possibility of insulinoma cannot be ruled out even if the IRI <0.3 [6], and in the study four people among the 22 patients with insulinoma are with the IRI <0.3. Therefore, to provide more reference parameters for the qualitative diagnosis of insulinoma, it is better to combine with the determination of the fasting blood glucose, the fasting insulin, the fasting C-peptide and the calculation of respective IRI.

It is relatively easy to make a qualitative diagnosis of hypoglycemia when the symptom is demonstrated as typical Whipple’s triad. Clinically, however, the symptoms of hypoglycemia for patients with insulinoma are more complicated and volatile, which include the non-specific autonomic nerve symptoms like profuse sweating, palpitation and trembling, as well as the central nervous system disorders like insanity, behavioral abnormality, personality change, epilepsy, narcolepsy, lethargy, coma [7–10]. The chronic patients can even suffer a remarkable reduction of blood glucose without any clinical symptoms [11, 12]. According to the study of Ding Bo et al. [13], more than half of the patients with insulinoma suffer from hypoglycemia at night and among the 22 patients with insulinoma in this study, there are 11 of them suffering the lowest blood sugar at night. Governed by the vagus nerves, the symptoms of hypoglycemia at night are still more unconspicuous. The traditional fingerstick glucose monitor only provides snapshots of plasma glucose, so reducing its reliability for quantification of hypoglycaemic episodes, especially at night. The traditional glucose examination is unable to discover the atypical hypoglycemia and the hypoglycemia at night timely. The continuous glucose monitoring system, however, can indirectly reflect the level of blood glucose by monitoring the glucose concentration of interstitial fluid of the subcutaneous tissues with a glucose sensor. Capable of offering the information about the blood glucose in all day, its major advantages lie in the fact that it is able to find out the hyperglycemia and hypoglycemia that are beyond the reach of traditional glucose examination, such as post-meal hyperglycemia and asymptomatic hypoglycemia at night, and it can define the time, frequency, and extent of hypoglycemia, making up for the deficiency of traditional glucose examination, and providing new clues to preliminary diagnosis [14]. The emergence of real-time continuous glucose monitoring system equipped with alarming functions for determining hyperglycemia and hypoglycemia, in particular, is beneficial to the diagnosis of insulinoma, especially the atypical cases. Studies show that CGMS contributes to increasing the detection rate of hypoglycemia and the asymptomatic ones in particular [15–17]. Han et al. contend that the lowest blood glucose detected by CGMS is of diagnostic value to insulinoma. In this study, there is also a marked difference of the lowest blood glucose between the insulinoma group and the hypoglycemia group and the lowest blood glucose of patients in the former group is remarkably lower than that in the latter group of which the hypoglycemia is caused by other reasons. Due to the different occurrence mechanism of hypoglycemia, the patients with insulinoma have suffered more serious hypoglycemia out of the unstifling secretion of endogenous insulin. In addition, this study suggests that hypoglycemia is of vital danger to the patients with insulinoma because of the high number in occurrence, the seriousness in extent and the long period of disease, therefore it is imperative to have early diagnosis and early treatment.

Currently, foreign literatures have referred to such indexes as MG, SD, LI, HBGI/LBGI, GRADE, MAGE, M value, CONGA, MAG, and J-Index, which are correlated with and different from one another, and reflect various aspects of the glucose level and glucose fluctuation based on different calculations. MAGE is considered to be the most comprehensive index for assessing GV in the past, but the calculation method of MAGE neglects the glucose rise or fall by less than 1 SD, so it only reflects the glucose fluctuation with relatively big amplitude and thereby is not quite objective; M value, with the ideal glucose as the baseline, makes the logarithmic transformation of the glucose fluctuation deviating from the baseline and then takes the average, it is a calculation which puts more emphasis on the deviation of hypoglycemia; LBGI is similar to M value, analysing the frequency and extent of hypoglycemia through the mathematical processing of blood glucose values; whereas CONGA-n represents the standard deviation of all current monitoring results against the monitoring results n hours ago, and it can evaluate the glucose fluctuation within the day in an accurate way, thus being more objective than MAGE [18]. It is a calculation which puts more emphasis on the deviation of hypoglycemia. This study suggests that although there are differences between patients with insulinoma and those with hypoglycemia caused by other reasons in SD and MAGE, they are not statistical enough, while the marked differences in CONGA, LBGI and M value are of statistical significance, therefore these are three indexes that are essential for making a clinical analysis of the glucose fluctuation characteristics of patients overwhelmed by hypoglycemia. Their more exact and sensitive descriptions of the extent and glucose fluctuation of hypoglycemia make it easier to tell clinically the insulinoma from hypoglycemia caused by other reasons. Based on the Youden and area under the ROC curve value, LBGI seems to be of the highest accuracy.

Because of the heterogeneity, the diagnosis and differential diagnosis of hypoglycemia is very complicated, determined mainly on clinical features, insulin levels, onset time and several tests. Glucose profile is only one part of these tests, and has not been studied broadly. Our study maybe provide a new clue to the hypoglycemia, and might be useful to identify insulinoma. Our study also has some limitations. First, the accuracy of CGMS sensor is more effective in elevated glycemic levels than hypoglycemic state, so it requires more frequent calibration by fingerstick tests. In our study, 4–6 fingersticks per day are needed to calibrate the CGMS for every person. However, there is still a need for more intensive research efforts in the future to develop robust, highly precise and specific CGMS, which will employ advanced algorithms to take into account the time lag between the concentration of glucose in the blood and the interstitial fluid. Second, due to the limited sample number, large-scale, multi-center study needs to be carried out for assessing the CGMS diagnostic value.
